# Free recall of semantically related words reveals similarity structure

**DOI:** 10.3758/s13421-026-01851-z

**Published:** 2026-02-03

**Authors:** Jeffrey C. Zemla, Nancy Linehan, Lynn J. Lohnas

**Affiliations:** Department of Psychology, Syracuse University, 765 Irving Ave, Syracuse, NY 13244 USA

**Keywords:** Free recall, Semantic memory, Episodic memory, Semantic networks

## Abstract

In free recall, semantic associations between studied items lead to clustering of those items. In prior work, the impact of these associations on recall has been assessed using measures that are independent of participant data. For example, the semantic similarity of *lemon* and *banana* can be estimated using a distributional semantic model (e.g., latent semantic analysis) and these estimates can be used to derive semantic clustering scores. We show that instead of using pre-existing estimates of semantic similarity, it is possible to estimate semantic similarity from the data itself. In one experiment, participants study categorized word lists that are either presented randomly or blocked (arranged by category). Using established and novel analyses, we find that temporal and semantic associations interact, but that semantic associations exert a predictable influence on recall order. We use this insight to develop a model for estimating pairwise similarity and a semantic network from free recall data. The estimated networks show high correspondence with both the category structure and a distributional semantic model (word2vec). Compared to word2vec, our model made more accurate predictions of clustering in free recall after controlling for temporal similarity, underscoring that similarity measures from different sources reflect different aspects of semantic information. We further validate the model using a large, pre-existing dataset (PEERS) of uncategorized free recall lists. The work presents a novel methodology that has many potential applications in the study of both episodic and semantic memory.

## Introduction

Memory retrieval is a complex cognitive process that is guided by mental associations, including those formed by conceptual similarity (relating to semantic memory) and proximity in time (relating to episodic memory). Though semantic and episodic memory are often characterized as separate domains of research, the two interact in substantial ways. One task that demonstrates this is free recall, which has provided a wealth of information regarding the cognitive processes and representations underlying episodic and semantic memory (Lohnas, 2024; Wixted & Rohrer, 1994). In a typical experiment, participants study a list of words presented sequentially on a display and then recall them in any order. The open-ended nature of free recall affords examining transitions between recalled items, assumed to reflect their underlying associations. The two most examined forms of transitions between words are based on temporal similarity (distance between words in the study list; Healey et al., 2019; Kahana, 1996; Polyn et al., 2009; Ward et al., 2010) and semantic similarity (Healey et al., 2019; Howard & Kahana, 2002; Pollio et al., 1969; Tulving & Pearlstone, 1966).

However, free recall studies that examine semantic associations typically face two major challenges. One challenge, as we review below, reflects the fluidity of temporal versus semantic associations, as these forms of organization may work with, against, or seemingly independent of one another. When semantic and temporal organization align (e.g., *apple* and *orange* are presented successively, thus promoting a strong semantic and temporal association between the item pair), the relative contributions of each type of association can be difficult to disentangle. A second challenge is that metrics of semantic similarity are often established by the researcher, rather than by participants. For example, a list of words to be studied might be constructed so that some words belong to the same category (Ward & Tan, 2023). Alternatively, the words themselves may be chosen at random and semantic similarity may be estimated using distributional semantic models based on word co-occurrence in a large text corpus (Howard & Kahana, 2002). Although these metrics are helpful in explaining participant behavior, they assume the underlying semantic similarity structure of the words (Manning & Kahana, 2012) prior to collecting data, rather than infer it from participant data. Thus, these metrics do not incorporate variability from the participant sample. Further, they do not vary across other variables which impact free recall performance, such as the composition of semantic similarity among studied words (Healey & Uitvlugt, 2019; Polyn et al., 2011) or even ordering among lists of the same semantic structure (D’Agostino, 1969; Puff, 1974; Weingartner, 1964). This is particularly problematic for similarity estimates derived from corpora, which in many cases are only weakly correlated with estimates from human data (De Deyne et al., 2016; Manning & Kahana, 2012; Suresh et al., 2023).

Measures of semantic similarity which are static across list designs and participants contrast with semantic similarity metrics that are estimated from psychological data itself using semantic memory tasks (De Deyne et al., 2019; Nelson et al., 2004; Suresh et al., 2023; Zemla & Austerweil, 2018). However, to our knowledge, no method for estimating semantic similarity from free recall data has been proposed. Here, we present a complementary approach to prior studies by exploring the inverse problem of estimating a model of semantic similarity from recall transitions.

In a single experiment, we asked participants to study and recall lists of categorically related words presented either in a random or organized (blocked) manner. We first review the temporal and semantic clustering properties of recall lists, and then propose a model for using temporal movements (lag in a study list compared to lag in a recall list) to estimate the similarity between word pairs. We demonstrate how this model can reconstruct the block category structure from recall data of randomized lists with high accuracy (without the need for any external measures of word-pair similarity) and predict which word pairs will be clustered together in recall.

To further validate this modeling approach, we then extend the model to lists without category structure using an existing dataset (PEERS; Kahana et al., 2024). Our results provide evidence that our model performs as well as a leading semantic similarity model for estimates of between-category semantic relationships. Lastly, we discuss several potential applications of our model including estimating semantic representations for groups and enhancing existing models of episodic recall.

### Semantic associations in free recall

Semantic associations between study items play an important role in free recall. For example, Howard and Kahana (2002) had participants recall lists of “unrelated” words and used latent semantic analysis (Landauer et al., 1998) to estimate the word-pair similarity for each recall transition. They found that transition probabilities increased with semantic similarity, despite the words being chosen to be unrelated so as to minimize the effects of semantic associations on recall.

Many studies have explored free recall of words drawn from a small number of discrete categories (e.g., a set of 15 words drawn from three categories). Typically, participants recall more words when the study list is blocked (grouped) by category as opposed to presented in a random order (D’Agostino, 1969; Puff, 1974; Weingartner, 1964), termed the blocked-random effect. Similarly, Huff et al. (2015) found that recall of blocked categories is superior to interleaved categories. While the organization of the study list influences recall, even the mere presence of semantically related words boosts recall. For example, randomized lists of thematically related words tend to be recalled better than randomized lists of unrelated words (Healey & Uitvlugt, 2019; Polyn et al., 2011).

Why is recall greater in lists with categorically or thematically related words? One possibility is that the retrieval of a word increases the retrieval strength of semantically related words through associative activation. This can be observed in the retrieval order of words during recall. When a study list is composed of words drawn from a small number of discrete categories, participants tend to cluster words from the same category together during retrieval (Bousfield, 1953). Clustering is also observed for semantically related word pairs, and the degree of clustering is related to the strength of association: Mayzner and Tresselt (1961) asked participants to recall a list of 30 words, including 15 stimulus words and 15 response words drawn from free association norms, presented randomly. The pairs were selected to vary in their association strength (i.e., frequency of response in the free association norms). For example, the stimulus word *salt* might appear in a study list with the response word *pepper* (high association strength), *taste* (medium strength), or *drink* (low strength). Recall clustering between such pairs increased as the mean strength of association between word pairs increased. Such clustering between words sharing stronger associations may then support performance on the task, consistent with findings that participants who exhibit greater clustering also have higher rates of recall (Patterson et al., 1971; Weingartner, 1964). Semantic associations affect not only the ordering of words in recall, but also response times. Many studies have demonstrated a pattern of response bursting for related words, i.e., smaller inter-item response times between word pairs of the same compared to different categories (Patterson et al., 1971; Pollio et al., 1969; Wingfield et al., 1998). These smaller inter-response times may be taken as evidence for stronger associations between semantically related words.

Much of this work focuses on the study of categorized lists or lists of associated word pairs that may draw a participant’s attention to the associative structure of the study lists. To some extent, this explicit knowledge can improve recall. For example, participants who re-organized a study list so that similar words were grouped had greater recall than those who chose to simply copy a study list (Burack & Lachman, 1996). Other work has suggested that the effect may be influenced by knowledge of category labels (Patterson et al., 1971; Tulving & Pearlstone, 1966). Additional mechanisms beyond associative activation may help to explain patterns of clustering and improved recall, such as rehearsal (Rundus, 1971; Ward & Tan, 2023).

### Temporal associations in free recall

Recall is also driven by the temporal order of words during study. For example, the distance between words in a study list (termed lag) is strongly related to the distance between words during recall. Recall of unrelated word lists demonstrate that people tend to transition between word pairs with small absolute lags, while transitions at larger absolute lags are increasingly less likely. In other words, the order of words during study bears a resemblance to the order of words during retrieval (Healey et al., 2019; Kahana, 1996; Polyn et al., 2009; Ward et al., 2010). These results provide strong evidence that participants form associations between items based on their temporal proximity in the list, whether directly through item associations (Kahana, 1996) or more indirectly such as through associations to a slowly drifting temporal context representation (Polyn et al., 2009). Like semantic associations, temporal associations may also reflect rehearsal processes, as items studied nearby in time are more likely to be rehearsed nearby in time (Laming, 2006; Rundus, 1971; Tan & Ward, 2000). The degree to which people transition between words with small lags (compared to large lags) is also positively correlated with recall accuracy (Healey et al., 2019; Sederberg et al., 2010).

### Interaction of semantic and temporal associations

Temporal associations and semantic associations can both aid recall, but in some cases they conflict. When a study list consists of categorically related words presented in a random order, increases in semantic clustering during recall result in less temporal clustering, and vice versa (Healey & Uitvlugt, 2019). In such lists, temporal clustering is greatly diminished compared to recall of unrelated word lists (Polyn et al., 2011). This result suggests that semantic associations can disrupt temporal clustering, as participants mentally re-organize items so that related words move closer together.

At the same time, temporal and semantic associations can also support one another. In lists of items drawn from different semantic categories, the temporal contiguity effect is higher for items from the same than from different categories (Polyn et al., 2011). However, when temporal and semantic associations align (e.g., presenting two semantically related words successively), assessing recall transitions alone may not be enough to disentangle contributions of episodic versus semantic memory.

### Estimating semantic similarity and networks from free recall data

Many of the studies described above explore the influence of semantic associations on free recall by examining whether semantically similar words are clustered together in recall. To measure this effect, a researcher must know which words in a study list are similar – often by estimating similarity between words using a model of semantic representation a priori (e.g., using latent semantic analysis to estimate word-pair similarity). In this paper, we propose an inverse approach: we aim to estimate a semantic representation (Cree & Armstrong, 2012; Jones & McRae, 2013; Kumar, 2021) from free recall data. In other words, we aim to estimate the semantic similarity of word pairs from the data, rather than analyze the data using a pre-existing semantic model. We start by estimating the similarity between each pair of words in a study list, and then use those similarity measures to construct a semantic network.

Semantic networks represent each unique concept as a node, and semantic similarity between concepts is represented by an edge between two nodes (Collins & Loftus, 1975). While semantic networks have not previously been estimated from free recall data, they have been estimated from many other psychological tasks including free association (e.g., De Deyne et al., 2019), triadic comparison (e.g., Chan et al., 1995), and semantic fluency (Christensen & Kenett, 2023; Zemla & Austerweil, 2018).

### The present work

In this paper, we present a novel method for estimating a semantic network from free recall data. Our method shares similarities with those used to estimate networks from semantic fluency (free listing of items from a category). In semantic fluency, people often cluster semantically similar items sequentially, for example, *cat*, *lion*, *tiger* (Bousfield & Sedgewick, 1944; Troyer et al., 1997) because retrieval of one animal (*lion*) activates related concepts in memory (*tiger*), increasing the probability that it will be retrieved next. As a result, proximity between words can be used to estimate similarity (Goñi et al., 2011). However, in free recall, we cannot rely solely on this as a proxy for similarity because it ignores the effect of temporal associations. To account for this, we present a model for estimating similarity using the change in lag (study list lag and recall list lag) for all possible word pairs. We show that the block structure of a word list can be reconstructed from free recall data, even when those words are studied in a random order.

To develop the semantic models, we conducted a novel experiment of free recall of categorized lists. We used categorized lists (as opposed to unrelated word lists) so that there is a prescribed, top-down semantic structure that we aim to recover from recall data to validate our model. We first examine temporal, semantic, and category clustering as well as how they interact using more traditional analyses.

We then present the semantic model, which provides new insights into episodic and semantic organization. To foreshadow our results, our model successfully recovered the categorical structure in the study lists with high specificity and sensitivity. When we compared our model to a distributional semantic model (word2vec; Mikolov et al., 2013) trained on the Google News corpus, we found a substantial correlation between the two models. Similarity estimates for the two models did diverge for between-category (i.e., low-similarity) word pairs, but we found that our model was more accurate in predicting semantically clustered pairs for free recall. We believe this underscores the idea that similarity estimates can vary across models in part because the role of similarity on behavior differs for various tasks (e.g., free recall vs. natural language) and in part for list design (e.g., blocked vs. randomized category order). At the same time, the results highlight that semantic models initially developed for semantic memory tasks nonetheless can elucidate organization in episodic memory tasks.

Finally, we apply the model to a large, open dataset of uncategorized lists to explore how the model performs using lists with less controlled semantic organization. Here as well, our model better accounted for semantic structure than word2vec. Taken together, these results reveal novel insights of the impacts and interactions of semantic and temporal similarity on free recall.

## Methods

### Participants

Four hundred participants were recruited from Prolific in exchange for one dollar. The target sample size was comparable to (or larger than) similar studies discussed in the *Introduction*. The median experiment completion time was 5 min and 13 s. Data from 279 participants were analyzed after exclusions. Participants were excluded if they recalled fewer than three valid words in a list (22 participants) or if they did not follow the instructions to press enter after each response (99 participants). Of the remaining participants, the average age was 39.17 (range 18–77) years. 219 identified as White/Caucasian, 33 as Asian, 18 as Black/African American, 23 as Hispanic/Latine, three as Native American/Alaskan Native, and two as Middle Eastern. 140 participants identified as female, 133 as male, and six as nonbinary. 273 participants (98%) reported that English was their first language.

### Materials

Study lists were constructed from two word pools used by Hong et al. (2024). Each word pool consisted of 15 nouns, five each from three categories.^1^ The first word pool contained vegetables (*asparagus*, *spinach*, *zucchini*, *beans*, *broccoli*), vehicles (*scooter*, *motorcycle*, *bike*, *tricycle*, *car*), and instruments (*banjo*, *guitar*, *cello*, *violin*, *clarinet*). The second word pool contained dwellings (*cottage*, *hut*, *cabin*, *bungalow*, *house*), leather goods (*shoes*, *boots*, *sandals*, *belt*, *saddle*), and botanicals (*lemon*, *grapefruit*, *banana*, *lime*, *dandelion*). A pseudo-randomized list and a blocked list were constructed from each word pool. The blocked lists were grouped by category, whereas the pseudo-randomized lists were randomized with the constraint that no more than two words from the same category appeared next to each other. See Table 1.

**Table 1:** Four word lists used in the study

Word pool	List type	Study list (in order)
**A**	**Random**	spinach, bike, violin, car, scooter, clarinet, beans, tricycle, motorcycle, guitar, broccoli, banjo, zucchini, cello, asparagus
**A**	**Semantic**	asparagus, spinach, zucchini, beans, broccoli, scooter, motorcycle, bike, tricycle, car, banjo, guitar, cello, violin, clarinet
**B**	**Random**	house, grapefruit, dandelion, boots, lemon, saddle, cabin, cottage, shoes, hut, banana, lime, sandals, bungalow, belt
**B**	**Semantic**	shoes, boots, sandals, belt, saddle, cottage, hut, cabin, bungalow, house, lemon, grapefruit, banana, lime, dandelion

### Procedure

Following informed consent, participants completed two free recall trials (study-distractor-test cycles). Participants received one randomized list and one blocked list, and the order was counterbalanced across participants preserving the order of the word pools across participants. After exclusions, 143 participants received a randomized list followed by a blocked list (A-random, B-semantic), while 136 participants received a blocked list followed by a randomized list (A-semantic, B-random). During the study phase, participants passively viewed a word list. Words were presented one at a time in all capital letters for 1.2 s followed by a 0.2-s interstimulus interval (blank screen). After each list, participants completed a distractor task consisting of ten arithmetic problems on the same page, following the same procedure as Hong et al. (2024). Each problem entailed subtracting fractions where each fraction simplified to an integer between 1 and 12. Participants selected the answer to each problem (an integer between 1 and 4) with a key press. After 30 s, the page auto-advanced regardless of accuracy. The purpose of the distractor task was to add a short delay between study and recall, and to minimize rehearsal by having participants engage in a task that requires working memory. Participants were not excluded based on performance in the distractor task. Participants were then prompted to recall as many words from the list as they could remember in any order by typing and pressing enter after each word. Participants were required to press enter so that the word would disappear from the screen. After 60 s, the page auto-advanced regardless of how many words were recalled. Participants repeated this process for each list (study list one, distractor trials, recall list one, study list two, distractor trials, recall list two). At the end of the experiment participants entered their demographics.

## Results

### Recall accuracy

**Fig. 1:**
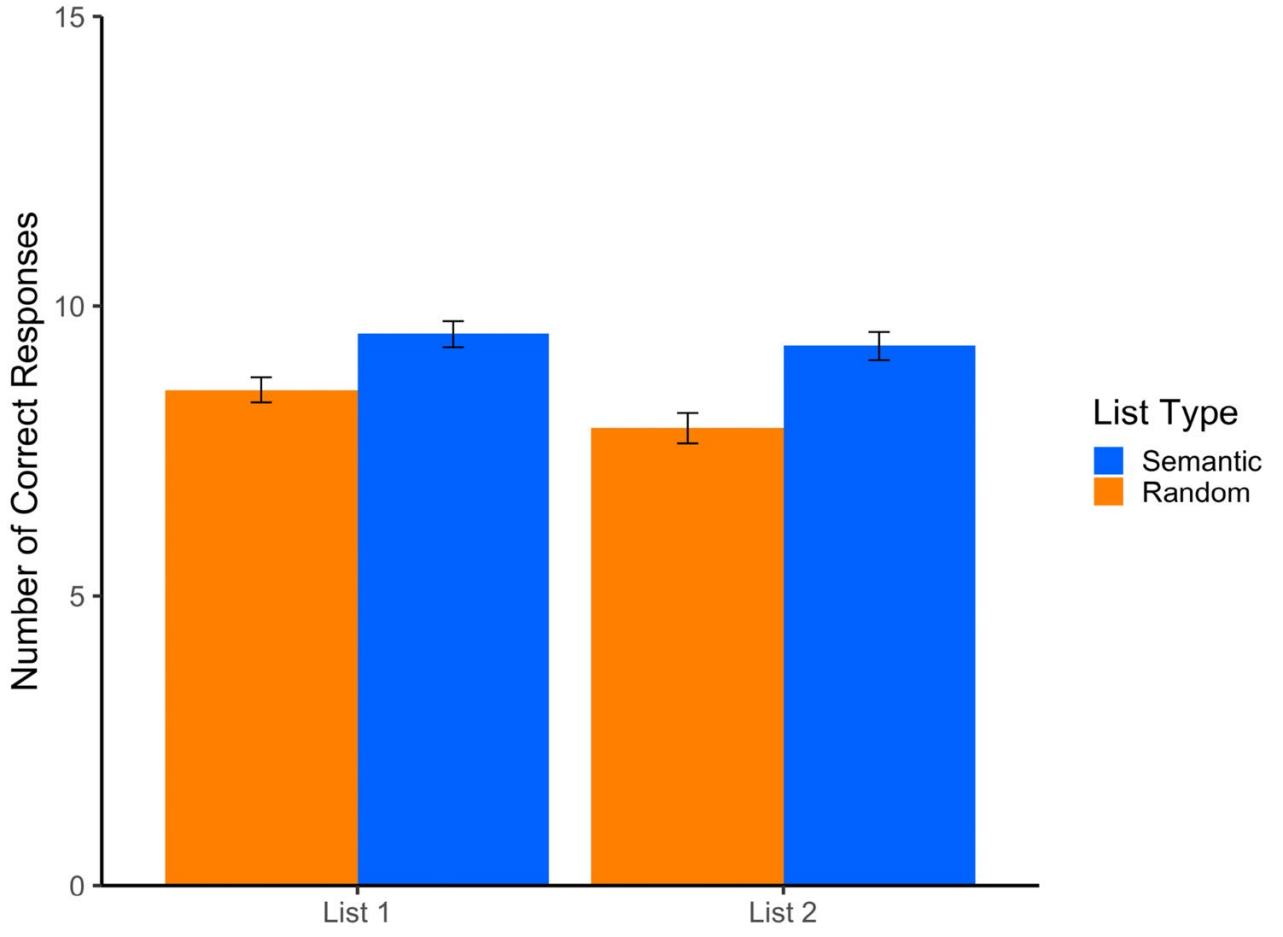
Participants correctly recalled more words in the semantic condition than the random condition. Error bars indicate standard errors

The data were pre-processed to correct spelling errors (406 in total). Spelling corrections were done through manual inspection by one of the authors, blind to condition. A list of spelling corrections is available in the Electronic Supplementary Material. Participants correctly recalled an average of 8.82 words (out of 15). A mixed-effects linear regression model was fit to the data with list number (one or two) and list type (semantic or random) and their interaction as predictors, plus a random intercept for participant. Participants recalled more words in list one than in list two, *M*_List1_ = 9.02, *SD*_List1_ = 2.64, *M*_List2_ = 8.62, *SD*_List2_ = 3.02, *t*(435.9) = −1.99, *p* =.047. Replicating the blocked-random effect, participants also recalled more words when the lists were semantically blocked compared to randomized lists, *M*_semantic_ = 9.41, *SD*_semantic_ = 2.76, *M*_random_ = 8.23, *SD*_random_ = 2.80, *t*(435.9) = 2.90, *p* =.004. There was no interaction between list number and list type (*p* =.43). See Fig. 1.

Errors were divided into four categories: extra-list semantic intrusions (words not presented but related to others on the study list^2^), extra-list non-semantic intrusions, and prior-list intrusions (words from study list one recalled during list two), and repetitions of the same word more than once in the same list. Intrusions were not common, accounting for just 3.3% of the data (115 extra-list semantic intrusions, 35 extra-list non-semantic intrusions, and 29 prior-list intrusions). Mixed-effects regression revealed that extra-list non-semantic intrusions were more common in list two than list one, *M*_List1_ =.04, *SD*_List1_ =.23, *M*_List2_ =.09, *SD*_List2_ =.36, *t*(544.1) = 2.55, *p* =.011. However, the number of extra-list semantic intrusions did not vary by list number (*p* =.78) or list type (*p* =.61), and the number of prior-list intrusions did not vary by list type (*p* =.16). Participants occasionally repeated the same word twice (repetitions) during the recall phase, accounting for 4.9% of the data (261 in total). However, their prevalence did not vary by list number (*p* =.95) or list type (*p* =.57).

### Category clustering

We assessed whether participants grouped words from the same category together during recall using category clustering scores. Category clustering was calculated for each list as the proportion of within-category transitions, given that transitions to both within- and between-category items remain possible (i.e., at least one item from the same category and one item from a different category have yet to be recalled). A score of 1.0 indicates perfect category clustering for the recalled words. For this analysis and subsequent clustering analyses, we collapse across list number because we found no significant interactions between list number and condition. Category clustering was significantly greater in semantically blocked lists than randomized ones, *M*_random_ =.46, *SD*_random_ =.24, *M*_semantic_ =.63, *SD*_semantic_ =.21, *t*(278) = 9.84, *p* <.0001 by paired *t*-test. Category clustering scores were positively correlated with the number of correctly recalled items in both semantic lists, *r*(277) =.33, *p* <.0001, and randomized lists, *r*(277) =.28, *p* <.0001.

For both semantic and random conditions, we also tested whether participants had category clustering scores significantly greater than chance. What constitutes chance depends on the set of items recalled by the participant. For this reason, we used resampling to estimate chance category clustering for each recall list. The set of items recalled by the participant was shuffled 1,000 times and the category clustering score was calculated each time. These were then averaged to form a chance category clustering score for the participant. We then calculated a difference score equal to the actual category clustering score minus the chance category clustering score. Significant category clustering (difference scores greater than zero) was observed in both randomized lists, *t*(278) = 11.1, *p* <.0001, and semantically blocked lists, *t*(278) = 25.6, *p* <.0001.

The above result demonstrates that participants cluster words by category more than chance in both conditions. However, when using a continuous dependent measure, it is always possible for a significant result to be driven by a small number of individuals who show a large effect, even if most participants show no effect. We thus conducted an additional analysis to check whether *most* participants show category clustering greater than chance. This was the case for the random condition (71.3% of participants, *p* <.0001 by binomial test), but even more so for the semantic condition (90.7% of participants, *p* <.0001 by binomial test).

### Semantic clustering

We calculated a semantic clustering score for each recall list, a measure that is conceptually related to the category clustering score but uses a continuous measure of semantic similarity between each word pair. For the purpose of calculating semantic clustering scores, similarity for each word pair was estimated using word2vec. In brief, word2vec estimates can generate a single number estimate of pairwise semantic similarity based on the predictability of words in a text corpus, and here we use estimates derived from the Google News corpus (Mikolov et al., 2013; https://code.google.com/p/word2vec/).

To calculate the semantic similarity scores, we first extracted the list of valid transitions, i.e., all transitions that do not include an intrusion or repetition. Next, for each transition, we generated a list of possible transitions, which includes all of the words that the participant *could* have recalled without error: words that were studied but the participant had not recalled up until that point. The clustering score for this transition is defined as the proportion of these possible transitions with semantic similarity less than the semantic similarity for the actual transition (Polyn et al., 2009). Thus, if the actual transition had the highest possible semantic similarity value of all possible transitions, then this would yield a proportion of 1.0. The semantic clustering score for a list is the average of these proportions across all transitions. If all transitions were among items with the highest possible semantic similarity, this would yield a score of 1.0 and indicate perfect semantic clustering. A score of roughly 0.5 indicates no semantic clustering (i.e., no semantic organization in the recall list). This calculation is analogous to the temporal clustering score (Polyn et al., 2009), but using semantic similarity instead of the absolute value of study lag.

Semantic clustering was significantly greater in semantically blocked lists than randomized ones, *M*_random_ =.60, *SD*_random_ =.15, *M*_semantic_ =.70, *SD*_semantic_ =.13, *t*(278) = 8.23, *p* <.0001 by paired *t*-test. Semantic clustering scores were positively correlated with the number of correctly recalled items in both semantic lists, *r*(277) =.23, *p* <.001, and randomized lists, *r*(277) =.20, *p* <.001.

For both semantic and random conditions, we also tested whether participants had semantic clustering scores significantly greater than chance following the same resampling procedure used for category clustering scores. Significant semantic clustering was observed in both randomized lists, *t*(278) = 10.7, *p* <.0001, and semantically blocked lists, *t*(278) = 25.5, *p* <.0001. In the random condition, 72.8% of participants had semantic clustering scores greater than chance (*p* <.0001 by binomial test), while 92.1% of participants had semantic clustering scores greater than chance in the semantic condition (*p* <.0001 by binomial test).

### Temporal clustering

**Fig. 2:**
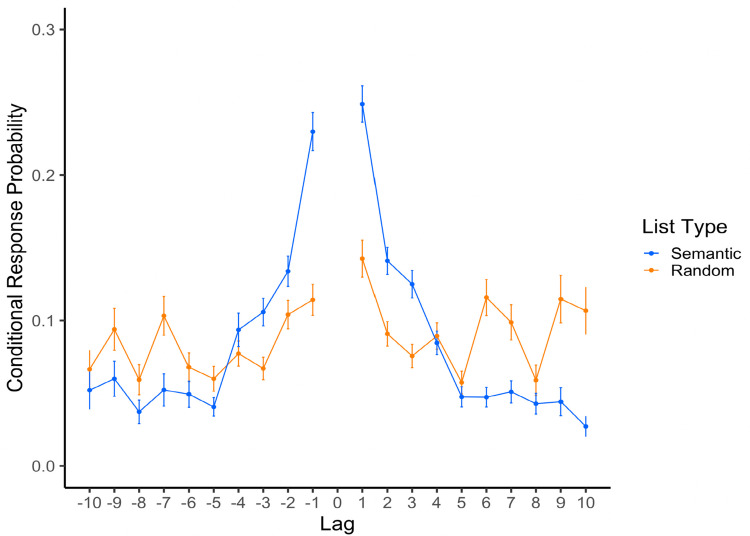
These lag-conditional response probability (CRP) curves (separated by condition) plot the conditional probability of transitioning to a response at each study lag, given that a valid transition to that lag is possible. Transitions to large study lags were less likely than to small study lags. Error bars indicate standard errors

We assessed whether, during recall, participants grouped words that were studied together. We did this in two ways: a lag-CRP curve and temporal clustering scores. First, a lag-CRP curve was constructed to visualize how the probability of recall depends on temporal lag in the study list, plotted separately for randomized lists and semantically blocked lists (Fig. 2). The curve shows the conditional probability of transitioning to a response at each lag, given that a valid transition to that lag is possible (see Kahana, 1996). The figure indicates that successively recalled words were more likely to have relatively small absolute lags than large ones. However, the effect *appears* to be larger for semantically blocked lists than randomized ones, evidenced by the greater probabilities at smaller absolute values of lag.

Second, we measured temporal clustering at the list level by calculating a temporal clustering score (Polyn et al., 2009) for each recall list. For each transition (excluding those that include intrusions or repetitions), the proportion of possible transitions whose lag is larger than the observed lag is calculated. The temporal clustering score is the average of these proportions over the entire list, with a score of 1.0 indicating perfect temporal clustering for the recalled words. Chance-level temporal clustering was calculated for each participant using the same resampling procedure used for category clustering and semantic clustering. Significant temporal clustering was observed in randomized lists, *t*(278) = 4.28, *p* <.0001, and in semantically blocked lists, *t*(278) = 23.42, *p* <.0001. Most participants (55.2%) had temporal clustering scores greater than chance in the random condition (*p* =.047 by binomial test), while 91.0% of participants had a temporal clustering score greater than chance in the semantic condition (*p* <.0001 by binomial test).

**Fig. 3:**
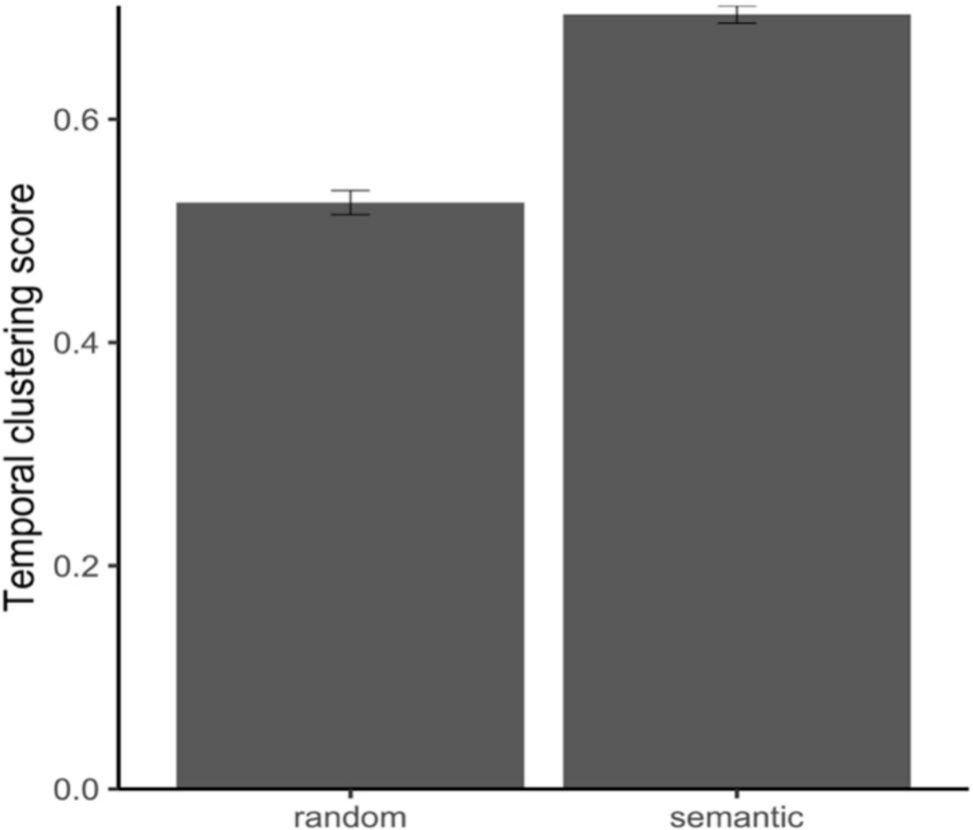
Temporal clustering scores were larger for semantically blocked lists than randomized lists. Error bars indicate standard errors. A box plot showing the distribution of temporal clustering scores in each condition is available in the Electronic Supplementary Material

Temporal clustering was significantly greater in semantically blocked lists than randomized ones, *M*_random_ =.52, *SD*_random_ =.18, *M*_semantic_ =.69, *SD*_semantic_ =.13, *t*(278) = 13.03, *p* <.0001 by paired *t*-test (Fig. 3). This aligns with a visual inspection of the lag-CRP curves (Fig. 2). Temporal clustering scores were positively correlated with the number of correctly recalled items in both semantic lists, *r*(277) =.17, *p* =.004, and randomized lists, *r*(277) =.13, *p* =.033. In addition, temporal clustering scores mediated the relation between list type and the number of correctly recalled items, *z* = 2.87, *p* =.004 by Sobel’s test.

### Interaction of temporal and semantic/category clustering

**Fig. 4:**
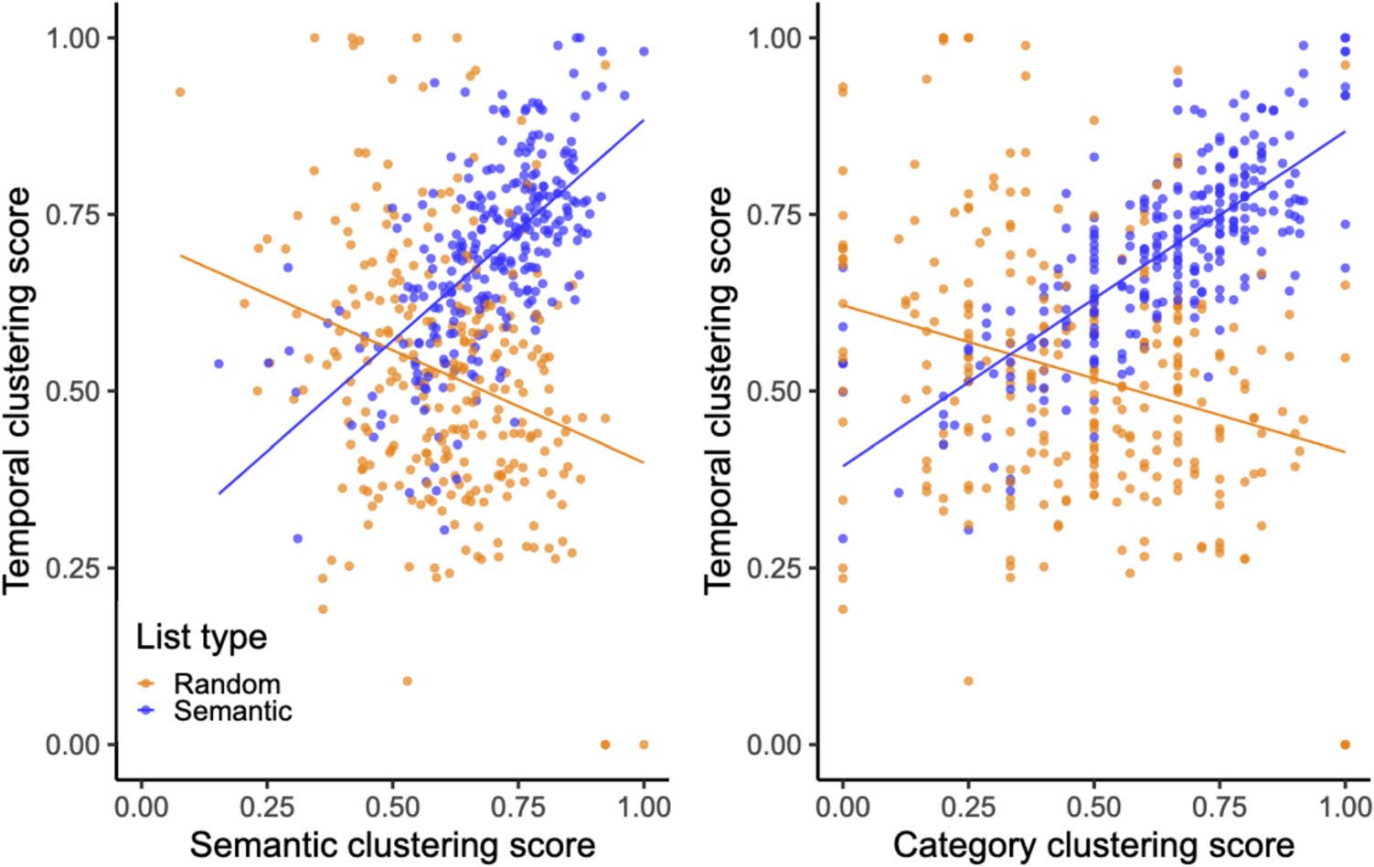
In semantically blocked lists (**blue**), temporal clustering scores were positively correlated with semantic clustering scores (**left**) and category clustering scores (**right**). In randomized lists (**orange**), these correlations were reversed

Having analyzed temporal and semantic clustering each in isolation, we next examined the interaction between these clustering types. First, temporal and semantic clustering were negatively correlated in recall of randomized lists, *r*(277) = -.27, *p* <.0001, but were positively correlated for blocked lists, *r*(277) =.62, *p* <.0001. Similar results were obtained when using category clustering scores instead of semantic clustering scores (*r* = -.28 and *r* =.77, respectively; both *p* <.0001). See Fig. 4.

**Fig. 5:**
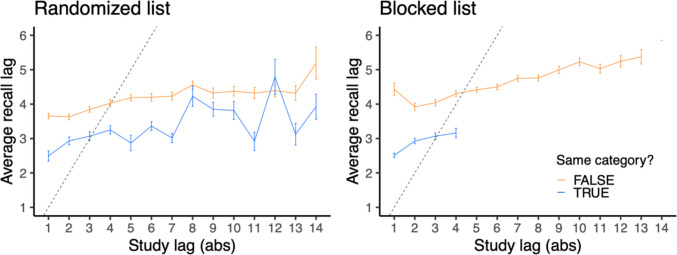
At nearly all study lags, the average recall lag was smaller for word pairs belonging to the same category than different categories, regardless of list type. The identity line (**gray dash**) is shown for comparison. Error bars indicate standard errors

Second, we analyzed the lag of word pairs in recall lists depending on whether the pair belonged to the same category. We call this *recall lag* to differentiate from the more traditional calculation of lag between words in a study list (*study lag*). For nearly all study lags, and regardless of list type, pairs from the same category were recalled with smaller values of recall lag than pairs from different categories. See Fig. 5. Note that unlike the lag-CRP curve, our analysis here uses the absolute lag (i.e., direction does not matter). Linear regression was used to predict the average recall lag from study lag, list type (blocked or randomized), pair similarity (same or different category), and all possible interactions. Study lag was positively associated with average recall lag, *t*(38) = 3.45, *p* =.001, and pairs belonging to the same category had smaller recall lag than pairs belonging to different categories, *t*(38) = 3.29, *p* =.002. No interactions were significant.

### Experiment discussion

In one experiment, we evaluated recall accuracy, temporal clustering, semantic clustering, and category clustering for categorized word lists that were arranged in either a pseudo-random or a blocked order. We found that blocked lists lead to greater recall than randomized lists, replicating the blocked-random effect. Moreover, we found that temporal clustering properties of blocked and randomized lists are very different. Recall of blocked lists shows very strong temporal clustering, while recall of randomized lists shows very weak (but still significant) temporal clustering. In other words, the order of words is fairly consistent between study and recall for blocked lists, but less so for randomized lists. Both randomized and blocked lists were semantically clustered. However, the association between semantic and temporal clustering reversed between list types: they were positively correlated in blocked lists and negatively correlated in randomized lists. We also found that for nearly all study lags, within-category word pairs appeared closer together (smaller recall lag) relative to between-category word pairs.

Collectively, these results suggest that the consistency of word order from study to recall depends in part on the semantic organization of the study list. Categorized word lists presented in a random order are substantially re-arranged during recall, reflecting the propensity for related word pairs to be recalled close together even when they are presented apart. Blocked lists had more consistency in word order from study to recall, reflecting a propensity for related word pairs to stay close together. This may also explain why temporal and semantic clustering are positively correlated in blocked lists, yet negatively correlated in randomized lists.

Nonetheless, interactions between temporal and semantic clustering can be challenging to disentangle, especially when semantically related items are also studied in close temporal proximity. In the next section, we describe a method for estimating the relatedness of word pairs from recall data, i.e., a semantic representation. In particular, we expected that semantically related word pairs could be identified by their change in lag relative to unrelated pairs, given Fig. 5.

### Model of similarity from free recall

#### Estimating similarity for pairs of words

Semantic representations have traditionally been derived from semantic memory tasks that have little to do with episodic memory (e.g., free association or semantic fluency). While free recall is more often thought of as an episodic memory task, the systematic influence of semantic associations on recall suggests that these associations can be recovered from the data. Here we apply a modeling approach to free recall data to capture relationships among semantically and categorically related items, as well as to demonstrate the utility of semantic networks to episodic memory tasks. Like clustering scores, our model assumes that items recalled in close proximity have stronger associations. Based on changes between study lag and recall lag, the model produced a representative network structure of semantic relatedness. Using data from the experiment, we estimated the semantic similarity of each pair of words in a study list using participant recall data and showed how this can in turn be used to form semantic networks.

We began by calculating the absolute recall lag between every possible combination of word pairs in each recall list (i.e., the absolute distance between two words in a recall list). For example, if a single participant recalls all 15 words (and no more), this results in 105 pairs from that participant (15 choose 2). Intrusions were not removed for the purposes of calculating the recall lag between two studied words. Similarly, repetitions were not removed for the purposes of calculating recall lag, but the recall lag for a given word pair was always measured using the first occurrence of a word.

We modeled the distribution of recall lags for any word pair using a truncated Laplace (double-exponential) distribution centered roughly on the study lag distance. For example, if a participant studies a list where two words have a study lag of three, we expect that the most likely recall lag between those two words in the recall data is roughly three, while recall lags larger or smaller than three are exponentially less likely. Mirroring the empirical data, this distribution accounts for the fact that small lag changes occur with high probability whereas large lag changes occur with lower probability. However, the center of this distribution needs to be adjusted to compensate for participants who recalled fewer words than were studied or, in a few rare instances, participants who recalled more words than were studied. This is because we are conditioning on the word pair being recalled (i.e., we can only calculate recall lags for words that were recalled in a list), and the expected recall lag between those words grows or shrinks depending on the number of words recalled. For example, if a participant recalls only five words, the recall lag cannot be larger than four, even if the study lag were larger. Specifically, we set the mean of the Laplace distribution to:

$${\mu }_{ijk}={S}_{ijk}+\left({n}_{k}-N\right)$$

where

μ_ijk_: is the mean of the distribution for word pair *i*, *j* and participant *k;*
S_ijk_: is the study lag between words *i* and *j* in the study list for participant *k*;
N: is the number of words in the study list (in our experiment, N is fixed to 15), and
n_k_: is the number of words recalled by participant *k* (including intrusions and repetitions).

Though each participant recalled two lists, we omit this to simplify the notation (we model blocked lists and randomized lists separately). The scale parameter of the distribution, which reflects the peakedness of the exponential, is fixed across all lists:

$$b=\frac{med\left(\left|{R}_{ijk}-{\mu }_{ijk}\right|\right)}{\mathit{log}(2)}$$

where

med(x): denotes the median of x;
R_ijk_: is the recall lag between words *i* and *j* in the recall list of participant k.

The distribution is truncated so that the maximum recall lag cannot exceed n_k_ and the minimum recall lag cannot be less than one. A normalizing constant is used so that the probability density integrates to one.

For each word pair, we calculated a semantic similarity metric using the cumulative distribution function of the Laplace distribution:

$${Sim}_{ij}=-\frac{\sum_{k}\mathrm{log}\left(P\left(lag\le \left.{R}_{ijk}\right|{\mu }_{ijk},b\right)+\delta \right)}{{T}_{ij}}$$

Sim_ij_ denotes the estimated similarity between word pair *i* and *j*, with higher values representing greater similarity. The numerator sums the log probabilities across all participants who recalled both *i* and *j*. In other words, this is the joint probability (in log space) of recall lags smaller than or equal to the observed recall lags for word pair *i* and *j*, given their study lags. T_ij_ is the number of participants who recalled both *i* and *j* and is used to adjust for the fact that some word pairs are recalled more often than others. To avoid log(0) errors, a small offset of δ = 1e-10 is added to each probability.

#### Semantic network estimation

Once Sim_ij_ is calculated for each word pair, a semantic network can be constructed. We did this separately for randomized study lists and blocked study lists and then compared the two. In a semantic network, each node represents a studied word and an edge between two nodes denotes that the words are estimated to be semantically related. A simple method for estimating networks is to estimate an edge between *i* and *j* whenever Sim_ij_ is greater than some threshold. However, a single threshold value is unlikely to work well for all possible study lists. In our experiment, the study lists were designed so that there are a large number of semantically related pairs, but this is not always the case for free recall data, where lists of unrelated words are often used. However, Sim_ij_ can be used to assess the *relative* similarity of words *i* and *j* compared to other word pairs from the same study list.

**Fig. 6:**
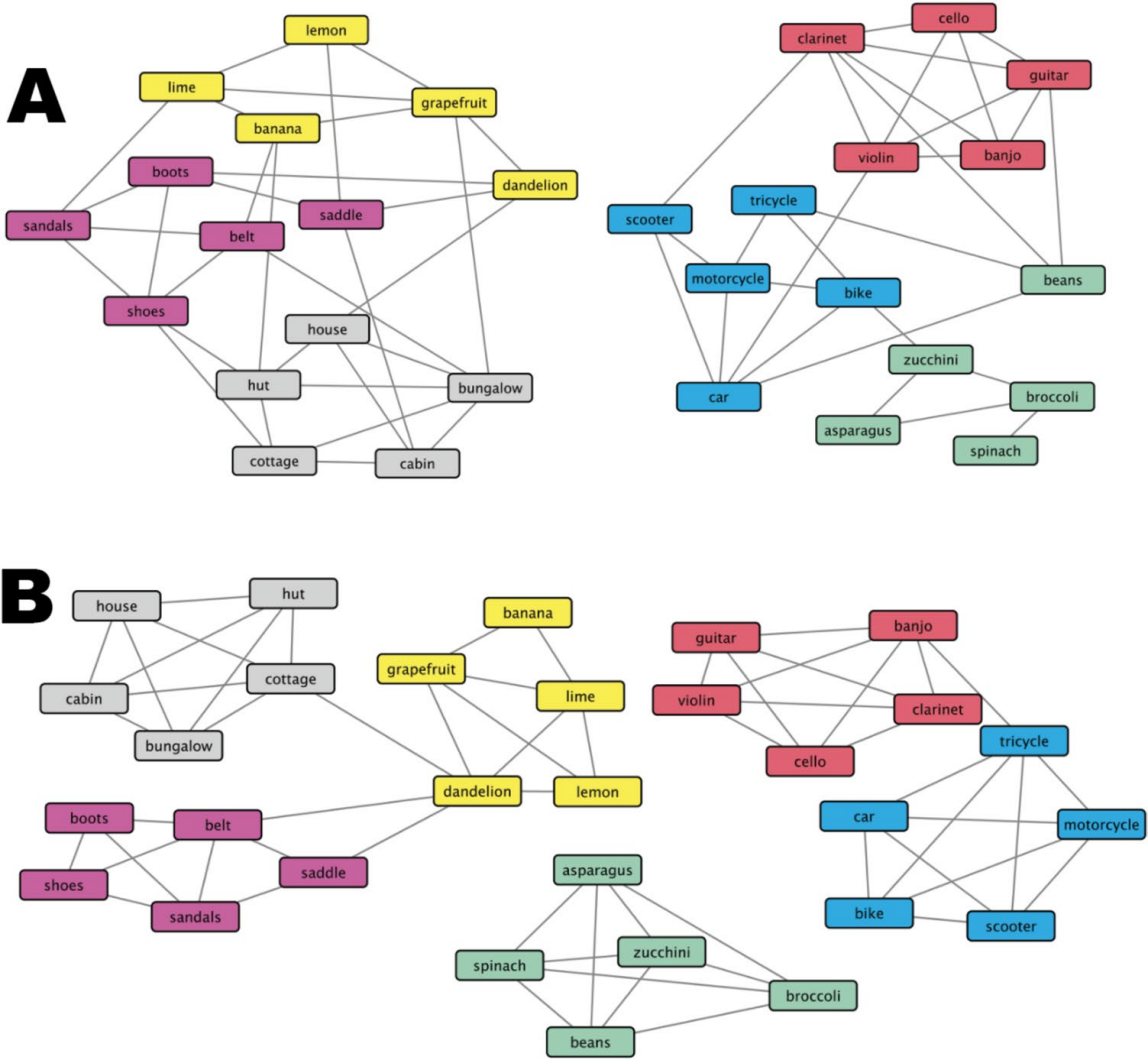
(**A**) Semantic networks estimated from the randomized study lists. (**B**) Semantic networks estimated from the blocked study lists. Nodes are color-coded by category for visualization

In the case of our experiment, we know the exact proportion of word pairs that are categorically related. Each study list contains five words from three distinct categories, so there are 30 categorically related pairs. Across two study lists, we expect 60 related pairs. Therefore we ordered the word pair Sim values from highest to lowest and used the highest 60 values to construct our networks. In the case of ties, word pairs were sorted alphabetically. The resultant networks are shown in Fig. 6.

Separate networks were constructed from randomized lists and blocked lists.^3^ We expected that networks generated from blocked lists would show greater agreement with the prescribed category structure, because this structure is already reflected in the word order of the study lists. Networks estimated from blocked lists represent a best-case scenario of what can be expected of the model, and can be used as a benchmark to compare to randomized lists. If one’s goal is to estimate semantic associations *independent of item order*, we do not recommend using recall data from blocked study lists. However, these networks can also be viewed as encoding semantic structure *in the context of* *item* *order*. Under this view, edges represent whether a word pair is similar enough to influence recall *given* their study lag, rather than in an absolute sense. This is notable because a semantic association between two words in a study list might exert no influence on recall if the study lag is sufficiently large.

### Model results

#### Comparing semantic networks to category structure

In Fig. 6, it is visually apparent that the estimated networks have edges between most within-category word pairs and fewer edges among between-category word pairs. The networks constructed from randomized lists have a sensitivity (true positive rate) of.68 and a specificity (true negative rate) of.87. The networks constructed from blocked lists have a sensitivity of.93 and a specificity of.97. The networks estimated from blocked lists have fewer false positives and false negatives than the networks estimated from randomized lists in part because the blocked study lists are already arranged by category and our analyses indicate a very strong preservation of this order in recall. As a result, recall lists partially reflect the structure that is already present in the blocked study lists. Interestingly, there are four adjacent word pairs in the blocked study lists that are not categorically related (house-lemon, cottage-saddle, broccoli-scooter, and banjo-car), yet the model was able to determine that these word pairs are not semantically related (no edge). These four pairs had an average Sim rank of 96.8 out of 210, well outside the top-60 threshold used to construct the networks.

**Fig. 7:**
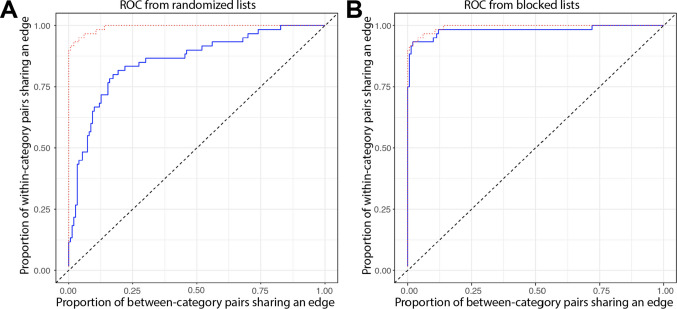
Receiver operating characteristic (ROC) curves (solid blue lines) show the trade-off between hits and false positives when constructing networks from pairs ranked by estimated similarity. For comparison, the dotted red line shows the ROC curve derived from word2vec and the dashed black line is the identity line (chance). (**A**) The ROC curve derived from randomized lists has an area under the curve of .856. (**B**) The ROC curve derived from blocked lists has an area under the curve of .981

For the above analyses it was assumed that the exact number of edges in the networks is known, but this is not always a reasonable assumption. A receiver operating characteristic (ROC) curve can be used to evaluate the trade-off between hits and false alarms for different threshold values if the number of edges is not known a priori. For these ROC curves, a hit reflects a correctly identified network edge for a within-category pair, whereas a false alarm reflects a network edge for a between-category pair. These are shown in Fig. 7 (blue lines). The area under the curve is .856 for the randomized lists, and .981 for the blocked lists. For comparison, chance performance is .5 and an ROC curve derived from word2vec similarity scores achieves an area under the curve of .994. So, the network estimated from blocked lists was comparable to word2vec, while the one formed from randomized lists was slightly worse. Nonetheless, the semantic networks maintain a high signal-to-noise ratio, far above chance, even for randomized lists.

The two networks constructed from randomized and blocked lists have a high degree of overlap, indicating that the model has fairly high reliability across different study list word orderings. Of the 210 possible edges, the networks disagreed on the presence of only 38 edges (18%). These edges were a mix of word pairs belonging to the same category (19) and different categories (19). The remaining 172 possible edges (82%) were either estimated to be an edge in both networks (41) or neither (131). Nearly all (168) agreed with the underlying category structure (i.e., within-category pair estimated to be an edge or between-category pairs estimated to not be an edge), while only four deviated from the underlying structure.

**Fig. 8:**
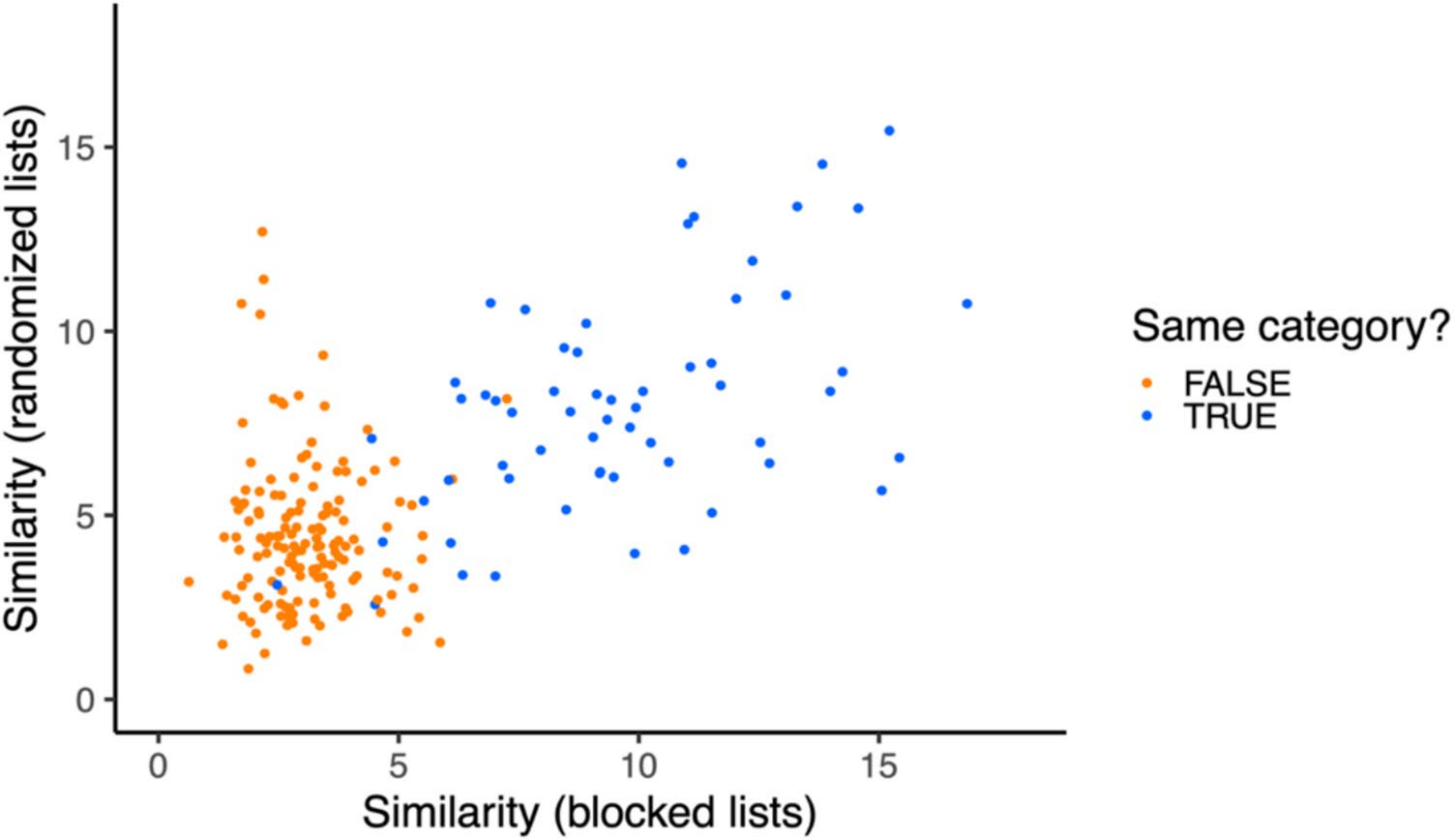
The similarity values derived from the blocked lists (**x-axis**) and randomized lists (**y-axis**) are shown for each word pair. Points are color-coded to indicate whether the pair belong to the same category or different categories

Our model allows us to compare how changes to temporal organization of categories (i.e., in randomized or blocked lists) moderates the influence of semantic associations on recall. Similarity estimates from randomized and blocked lists should be correlated to the extent that the estimates reflect stable associations across study list orders. Similarity estimates are directly comparable because the lists contained the same words, and varied only in word order. In general, Sim values derived from randomized and blocked lists are correlated, *r*(208) =.62, *p* <.0001. This is driven by a correlation of within-category word pairs, *r*(58) =.53, *p* <.0001. In contrast, the correlation of between-category word pairs was not significant, *r*(148) = -.03, *p* =.76. See Fig. 8. A plausible reason for this is that within-category word pairs systematically move closer to each other (in randomized lists) or stay close to each other (in blocked lists). Between-category word pairs have low similarity, and so these pairs may align more closely with study list order. In these cases, the similarity estimates may reflect temporal order, which is not correlated across list types. For example, it is likely not a coincidence that *beans* and *clarinet*, a between-category pair, have a study lag of one in the randomized list and are estimated to be joined by an edge in the semantic networks; they may stay close to each other in recall purely from their strong temporal association. In other words, while our aim is to disentangle semantic and temporal associations, false alarms in the networks often (though not always) have small study lags.

#### Comparing our semantic model to word2vec

Having established that the experimental data provide reasonable and stable estimates for our model, we next assessed whether our model aligned with an independent semantic model, word2vec pre-trained on the Google News corpus.

#### Similarity values

**Fig. 9:**
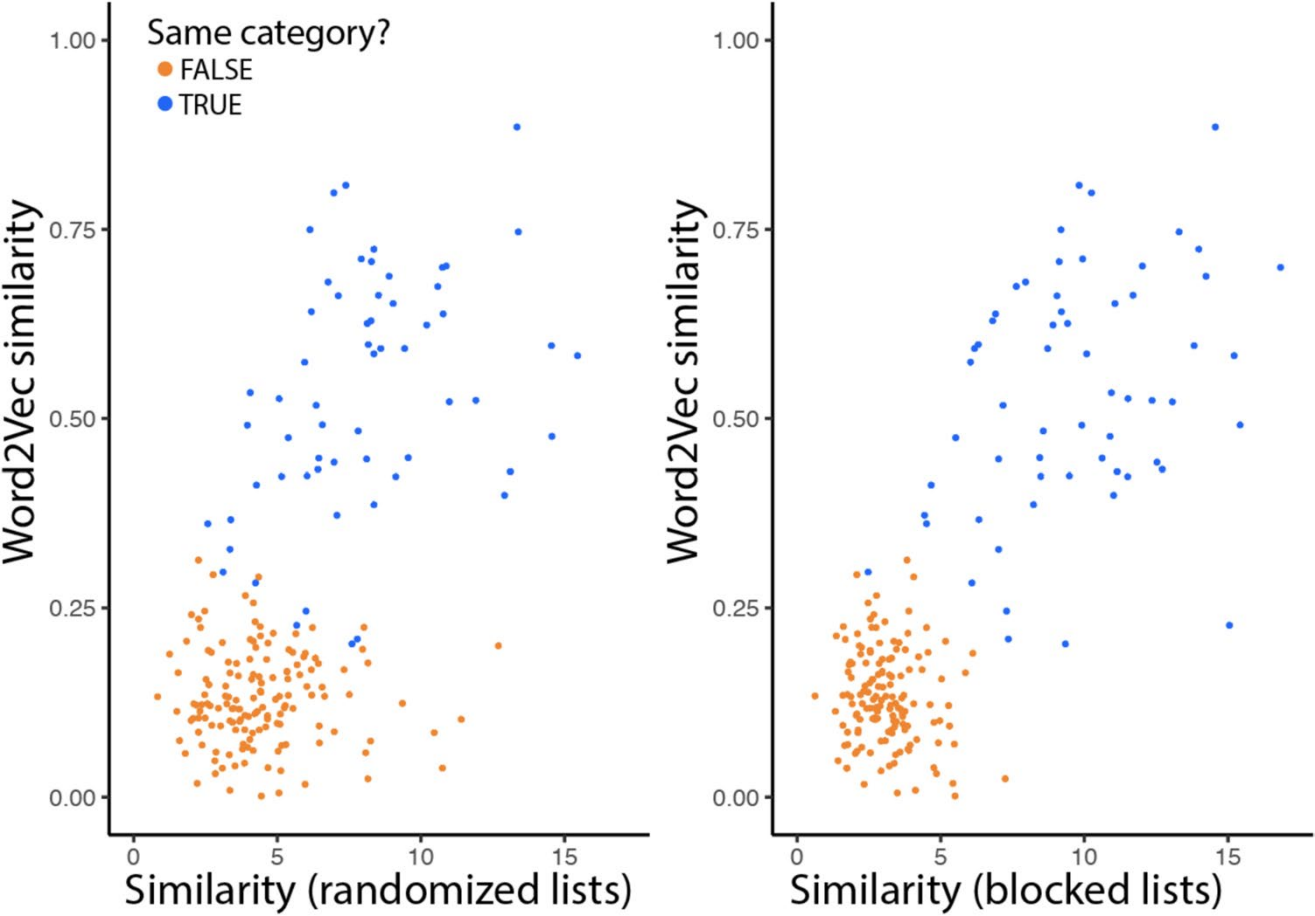
Word pair similarity derived from free recall data was correlated with similarity derived from word2vec. This correlation is driven primarily by words that share a category

Word2vec similarity was correlated with our semantic model similarity metric estimated from both the randomized lists, *r*(208) =.59, *p* <.0001, and the blocked lists, *r*(208) =.80, *p* <.0001. Again, these correlations were driven by within-category pairs, *r*(58) =.39, *p* =.002 for randomized lists and *r*(58) =.34, *p* =.007 for blocked lists. In contrast, the metrics were uncorrelated for between-category pairs, *r*(148) = -.02, *p* =.79 for randomized lists and *r*(148) = -.13, *p* =.12 for blocked lists. See Fig. 9.

#### Predicting semantic clusters in free recall

The significant correlations between the model’s similarity estimates and word2vec similarity suggest that the model boasts some of the same predictive power as word2vec. We next assessed whether estimates from our model were better than word2vec at predicting which word pairs would be clustered more than others in recall. Such a comparison underscores the idea that similarity estimates in both models are biased by their respective training data: since our model is trained on free recall data and word2vec is not, we expected that our model should be better at predicting semantic clustering in free recall.

First, we split the data into two halves by participant and used the model to produce two similarity estimates for each word pair (one estimate from each half). Second, for each half, we calculated the proportion of times each word pair had a recall lag of one, given that they were recalled in the same list. For example, in one fold of the data, *lemon* and *lime* were recalled 69% of the time with a lag of one (despite having a study lag of seven). We then used the similarity estimates obtained from one half to predict the proportions obtained from the other half. This process was performed separately for randomized and blocked lists.

**Fig. 10:**
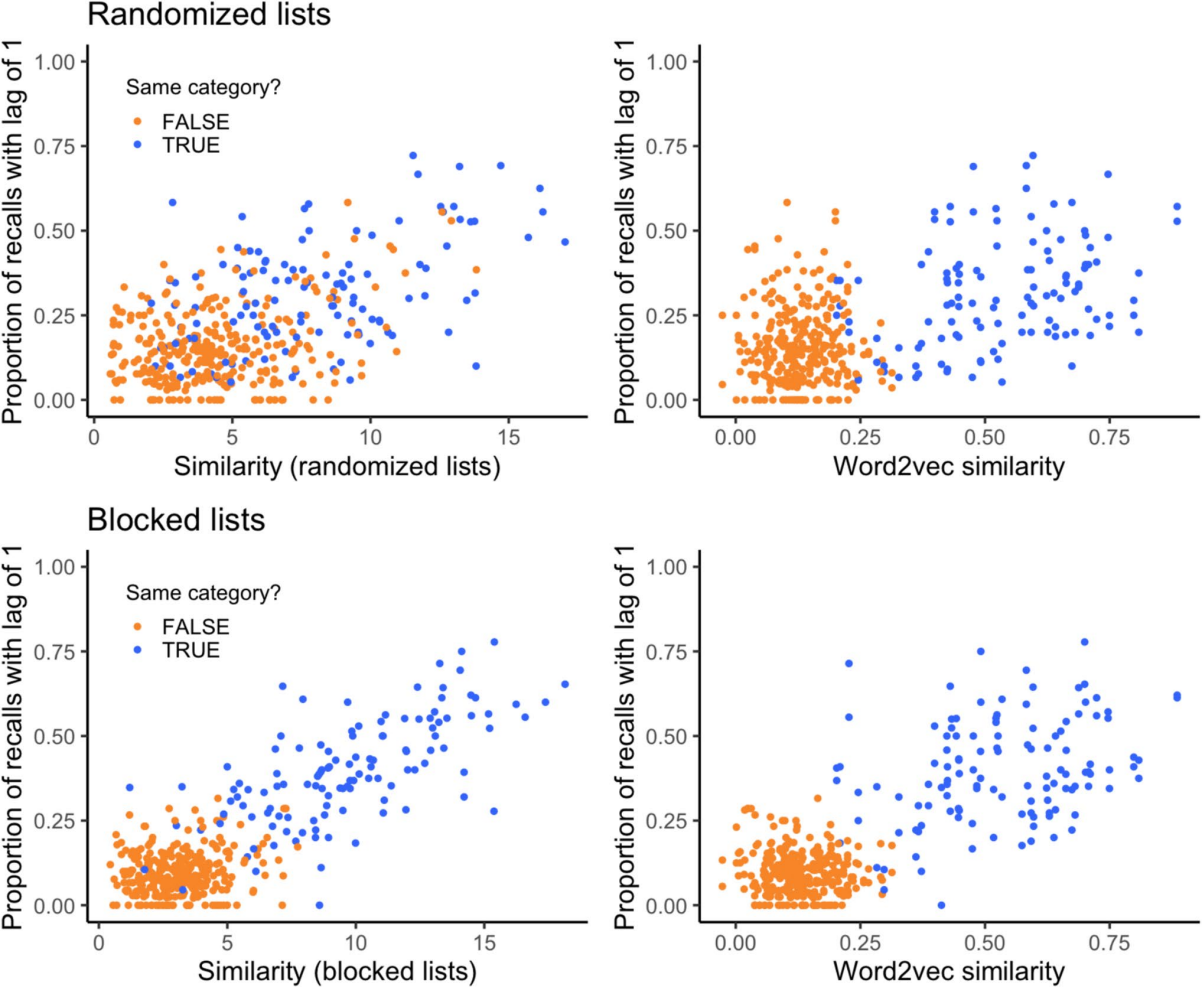
Split-half cross-validation analyses were performed separately for randomized lists (**top row**) and blocked lists (**bottom row**). Both similarity estimates from our model (**left column**) and word2vec (**right column**) were correlated with the proportion of times a word pair was recalled with a lag of one, though on average our model estimates were a better predictor

The results are visualized in Fig. 10. For randomized lists, the model estimates significantly predicted the proportion of times the words were recalled with a lag of one, *r*(418) =.54, *p* <.0001. Word2vec similarity was also correlated with proportions to roughly the same degree, *r*(418) =.51, *p* <.0001. Multiple regression was used to predict the proportion of lag-1 recalls from our model’s similarity estimates, word2vec, and study lag.^4^ All three factors were significant: model similarity, *t*(416) = 7.45, *p* <.0001, word2vec similarity, *t*(416) = 7.47, *p* <.0001, and study lag, *t*(416) = −3.97, *p* <.0001, multiple R^2^ =.386. The regression model was re-run separately for within-category and between-category pairs. For within-category pairs, both similarity measures were significant while study lag was not: model similarity, *t*(116) = 4.41, *p* <.0001, word2vec similarity, *t*(116) = 2.43, *p* =.017, and study lag, *t*(116) = −1.84, *p* =.069. For between-category pairs, word2vec similarity was not significant (*p* =.64), while model similarity, *t*(296) = 4.81, *p* <.0001, and study lag, *t*(296) = −4.16, *p* <.0001, were significant.

Similar results were obtained for semantically blocked lists: the proportion of lag-1 recalls correlated with both model estimates of similarity, *r*(418) =.83, *p* <.0001, and word2vec similarity, *r*(418) =.76, *p* <.0001. In a multiple regression model, model similarity estimates were significant, *t*(416) = 13.84, *p* <.0001, as well as word2vec similarity, *t*(416) = 6.71, *p* <.0001, and study lag, *t*(416) = −5.02, *p* <.0001, multiple R^2^ =.747. For within-category pairs, model similarity was significant, *t*(116) = 6.30, *p* <.0001, as was study lag, *t*(116) = −3.06, *p* =.003, and word2vec similarity was nearly significant, *t*(116) = 1.96, *p* =.053. For between-category pairs, model similarity was significant, *t*(296) = 2.72, *p* =.007, as was study lag, *t*(296) = −4.07, *p* <.0001, but word2vec similarity was not, *t*(296) = −1.32, *p* =.19.

To establish the reliability of the model, we also calculated correlations between both folds of the data (i.e., split-half reliability). Similarity estimates from randomized lists were significantly correlated, *r*(208) =.54, *p* <.0001. These correlations held for both within-category pairs, *r*(58) =.47, *p* =.0001, and between-category pairs, *r*(148) =.32, *p* <.0001. Similarity estimates from blocked lists were also significantly correlated, *r*(208) =.56, *p* <.0001. Again, this held for both within-category pairs, *r*(58) =.68, *p* <.0001, and between-category pairs, *r*(148) =.18, *p* =.027.

These results underscore the predictive power of our model. Across all list types and pair types, model similarity contributed significantly to predicting recall transitions. The predictive power of word2vec similarity was generally weaker, especially for between-category pairs. Whereas within-category pairs reflect salient semantic categories that are probably shared across participants, transitions among between-category pairs may not reflect shared associations. Thus, these pairs may pose more of a challenge to word2vec (and, indeed, any similarity metric).

### Application of the model to a large dataset of uncategorized lists

Having validated our model on categorized lists, we explore a potential application of our model by applying it to a much larger corpus of free recall data of uncategorized lists. We use data from the open PEERS dataset Experiment 4 (Aka et al., 2021; Cohen & Kahana, 2022; Kahana et al., 2024; Li et al., 2024). We summarize the experimental design here and refer the reader to Kahana et al. (2024) for more details. Ninety-five participants completed 23 sessions^5^ of free recall and each session contained 24 trials. Each trial consisted of 24 words presented sequentially (1,600 ms each, variable interstimulus interval 800–1,200 ms), followed by a 24-s distractor task (solving basic addition problems), followed by a recall phase. Half of the trials were preceded by an additional distractor task. In total, 576 unique words (24 x 24) were presented in each session. These same 576 words were repeated across each session, but the order of words was pseudo-randomized. As such, each word appeared in multiple contexts (both within and across participants). Participants were able to take a short break every eight lists.

Each word list was constructed such that eight words were selected at random from the word pool and an additional eight word pairs (16 words) were chosen based on semantic similarity. This was done by dividing all possible word pairs into five evenly spaced bins of semantic similarity (based on a word association space; Steyvers et al., 2005), and two pairs were selected from each of four bins. At least one pair was presented contiguously, and one pair was presented with at least three intervening items.

**Fig. 11:**
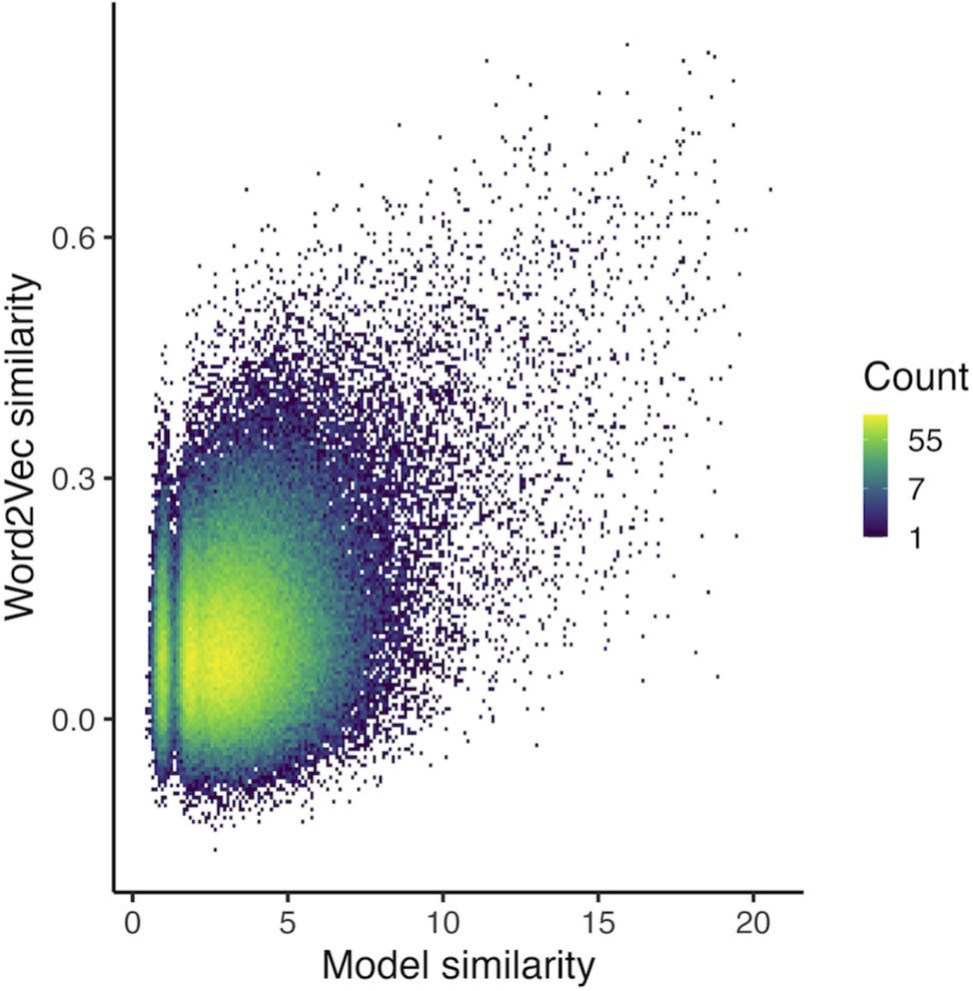
Across 165,600 word pairs, estimates of similarity derived from word2vec (**y-axis**) and our own model (**x-axis**) were significantly correlated

We estimate the semantic model in an identical way as before, resulting in similarity estimates for 165,600 word pairs. We compared these estimates to those from word2vec and found a moderate correlation, *r*(165598) =.325, *p* <.0001. Figure 11 illustrates this correlation as a heat map. This correlation appears to be driven by mid- and high-similarity pairs, similar to how in our experiment the correlation was driven by within-category pairs. This can be seen visually in Fig. 11 in the large mass of low-similarity words (green/yellow blob in the bottom left), but can also be observed by assessing how the correlation changes after removing word pairs below a given threshold of word2vec similarity. Starting around the lowest similarity (˗0.15) and increasing in increments of 0.01, the correlation monotonically increases until a threshold of 0.25 (figure available in Electronic Supplementary Material); the correlation between word pairs with a word2vec similarity of at least 0.25 is *r*(11622) =.511, *p* <.0001.

## Model discussion

Development of semantic models helps to elucidate how semantic and temporal similarity during initial encoding inform subsequent free recall. The semantic networks estimated from both list types reflect the underlying category structure, yet the blocked lists supported more faithful representation of the prescribed categories. This finding aligns well with a positive correlation between temporal and semantic clustering scores in blocked lists yet a negative correlation between these clustering scores in randomized lists. The blocked list model serves as a better benchmark because within-category items were studied nearby in time, thus aligning temporal and semantic similarity. However, the model from randomized lists had much overlap with the blocked list model, underscoring shared semantic representations derived from both list types. On its own, the randomized list model served as a good approximation of the underlying semantic structure. The similarities between these models and the word2vec model also underscore the predictive power of models developed from participants’ recall sequences. Thus, overall the models revealed novel tradeoffs between semantic and temporal representations, as well as the benefits of using participants’ own recall sequences to estimate the underlying semantic networks informing recall.

We also demonstrate a direct application of our model: predicting which word pairs are likely to be clustered together in recall. Cross-validation analyses showed that our model was able to predict clustering behavior comparable to or better than word2vec similarity (despite being trained on far less data). Both similarity measures were significant in a multiple regression analysis. Notably, estimates from our model significantly predicted semantic clustering in between-category pairs while word2vec similarity did not, despite controlling for study lag (temporal similarity). This suggests that the two sources may encode partially distinct information, though our analyses do not indicate what those specific differences might be. Another possibility is that, since both estimates have noise, word2vec similarity is a more valid predictor for some pairs while our metric is more valid for other pairs.

In addition, semantic similarity estimated from a large dataset of uncategorized lists was found to correlate with word2vec. One reason this is notable is because, unlike in our experiment, study lag between word pairs varied across participants. As a result, the estimates do not reflect the interaction of semantics and any particular study order, demonstrating that the model can also be useful to those who are interested in estimating semantic associations that are relatively stable across study order. In addition to generalizing across different study lags, these results also generalize the predictive power of semantic models in free recall for lists with or without a categorical structure. Categorized lists provide a more salient semantic structure, and thus arguably provide a more straightforward modeling task than lists of “unrelated” words. Further, the latter type of semantic manipulation reflects a more standard manipulation in free recall.

## General discussion

In one experiment, we replicated the block-random effect and found that the temporal clustering properties in recall depend on the list type (randomized or blocked). Randomized lists showed weaker temporal clustering, reflecting the re-arrangement of words during recall, while blocked lists showed strong temporal clustering, reflecting a stronger preservation of word order during recall. Further analyses on word pairs revealed that for any given study lag, word pairs from the same category tend to be recalled with smaller recall lag than word pairs from different categories. These properties motivated the construction of a model for estimating semantic similarity from recall data. We estimated semantic networks from the recalled lists and, using the category structure as a ground truth, found that the networks had both high sensitivity and high specificity. Additionally, we found our model made good predictions about semantic clustering (how often each word pair would be clustered together). We found that for this application, our model generally made better predictions than word2vec. Notably, our model significantly predicted clustering of between-category word pairs while word2vec did not (in a multiple regression model), even after controlling for temporal order in the study list. Finally, we explored an application of this model to a large dataset of uncategorized word lists and found it to adequately capture semantic associations among word pairs (particularly those with high similarity).

At a surface level, our results are not entirely surprising: similarity influences recall order, and therefore it makes sense that recall order could be used to recover similarity. However, this is not guaranteed to be true with our experimental design. Computational models in psychology often seek to make inferences about unobservable mental states, and often they fail. The reasons vary, but can include failure to account for moderating factors or collecting insufficient data. For example, we obtained 420 pairwise similarity estimates from 558 recall lists (and, for the PEERS dataset, 165,600 estimates from 52,440 recall lists). If the association between lag and similarity were much weaker, it is conceivable that our results would have been unreliable unless we collected more data. The model itself contains no free parameters, and does not account for individual differences in cognition that could affect recall (beyond what is reflected in the recall lists). However, we do believe there is room for improvement, and our results set a benchmark for future research.

Our modeling approach reveals similarities to and differences from word embedding models, a valuable methodological tool in psychological research. Here, we have used one such model – word2vec – as a benchmark for comparison. Despite their success, word embeddings differ from psychological data in several key ways. Foremost, word embeddings are models of meaning through language, not mental representations (Contreras Kallens & Christiansen, 2025). Embedding models are trained on millions or billions of documents that were never designed to probe how conceptual similarity affects mental processes. As a result, researchers often treat word embeddings as invariant to the mental processes being modeled. In contrast, various behavioral tasks used to estimate semantic similarity (such as pairwise ratings, semantic fluency, and triadic comparison) reflect the many different ways people use semantic knowledge: pairwise similarity ratings may reflect the ability to bring to mind shared attributes, while similarity estimates derived from free recall may reflect the interaction between semantic and episodic memory. For example, in free recall, the probability that two *instrument* words will be recalled together will differ if they are studied far apart in a categorized list versus presented nearby and all other words are *fruit*. Of course, embedding models have been extremely influential in psychology in part because they *do* make good predictions about human similarity ratings. We are not arguing that researchers should stop using embedding models. Rather, our intent is to highlight that word embeddings are distinct from our model, and that one is not intrinsically better than another. Our model also presents several methodological opportunities, discussed below, which include: comparing semantic representations across groups, exploring semantic context effects, and incorporating estimates from our model into process-level models of free recall to improve behavioral predictions.

One promising research direction is to adapt our approach to derive participant-level semantic similarity estimates. Because language models like word2vec extract similarity based on large corpora of text, how to apply these models to individual participants is less straightforward (one would need to train the embeddings on text from a single individual). Yet participant-level networks have multiple benefits. Networks formed by aggregating data across participants can have different properties (e.g., connectedness, degree distribution) that do not reflect any one individual’s network (Morais et al., 2013). In addition, individual networks allow for analyses with covariates that are not possible with aggregate networks. For example, Zemla and Austerweil (2019) found that scores on the Mini-Mental State Exam (Folstein et al., 1975) were correlated with network structure in older adults with Alzheimer’s disease. Previous work has estimated such participant-level semantic networks from free association and semantic fluency data (but not free recall) in different ways. One is to collect a large amount of data per participant. For example, Morais et al. (2013) collected data from participants using daily 1-h sessions over 6 weeks. While this is certainly labor intensive, it is not infeasible. In fact, the PEERS dataset we analyzed – which uses the same set of study words for each of 23 sessions – might be suitable for this approach. A second approach is to use a smaller amount of data per participant, but compensate for this by using a hierarchical model (estimating multiple participant networks simultaneously) or incorporating a prior (Zemla & Austerweil, 2018, 2019).

A related application is to estimate semantic networks for specific groups of interest. Prior work suggests potential applications of this methodology. In particular, networks estimated from semantic fluency data and other tasks have proved useful for assessing psychological questions about the nature of semantic representations in individuals or groups. For example, they have been used to evaluate differences in semantic memory between healthy adults and individuals with Alzheimer’s disease (Zemla & Austerweil, 2019), older and younger adults (Wulff et al., 2022), highly creative individuals (Kenett et al., 2014), and children (Beckage et al., 2010). Estimating semantic networks from free recall provides some potential advantages over other tasks such as semantic fluency (Goñi et al., 2011), free association (De Deyne et al., 2019; Nelson et al., 2004), or triadic comparison (Chan et al., 1995). One advantage is that free recall constrains the set of concepts to a finite and arbitrary set of concepts (compared to semantic fluency or free association), mitigating methodological problems that arise from comparing networks of different sizes (Zemla, 2022). In addition, free recall provides an implicit estimate of semantic similarity, which may differ from explicit judgments made by participants in triadic comparison or pairwise similarity judgments. Ideally, these estimates should converge across different methods of elicitation (Suresh et al., 2023) to the extent that they capture core aspects of conceptual meaning that are stable across contexts (Rosch, 1973; Rogers & McClelland, 2004). Compared to word embeddings, our model is well suited to explore how mental associations might differ between groups (e.g., monolinguals vs. bilinguals) or depend on some experimental manipulation, which can be accomplished by using different datasets to estimate similarity. It is less clear how word embeddings could be used, since they are trained on text corpora such as Google News, rather than text from any specific population.

Ultimately, the choice to estimate semantic networks from pairwise similarity is an arbitrary one and not strictly necessary; our continuous similarity metric was significantly correlated with similarity from an independent source (word2vec), indicating some ability of the model to discern a more nuanced measure of similarity beyond the block category structure. However, the coarseness of the network representation may lead to improvements in reliability. In contrast to the continuous similarity metric (Sim), edges in our semantic network are binary, i.e., an edge denotes a semantically similar word pair and the lack of an edge denotes an unrelated word pair. This presents a more achievable goal, particularly given the categorical nature of the study lists in our experiment. One remaining challenge is disentangling semantic and temporal similarity when an unrelated word pair is both presented and recalled adjacently, which the model can misinterpret as greater semantic similarity. Further insights could be gained by exploring measures of uncertainty that attenuate confidence when the model has less information (i.e., fewer observations). For example, one could construct a confidence interval around similarity estimates that depends on the number of observations in the training set.

The most direct applications of our model may be to research in episodic memory, because the representations reflect how similarity influences memory retrieval. In particular, one can interpret the similarity of a word pair estimated from our model as reflecting the impact of similarity on recall sequences. This could be useful for researchers who are interested in whether the semantic similarity of a word pair is sufficient to have an effect on recall. This may be particularly informative when examining how semantic similarity interacts with temporal similarity or list composition (e.g., Healey & Uitvlugt, 2019; Howard & Kahana, 2002; Ward & Tan, 2023). Our results provide some initial evidence that our model is well suited to make predictions in free recall. Compared to word2vec, similarity estimates from our model were as good or better at predicting whether a word pair would be clustered in recall, and the two measures of similarity each explained unique variance. Prior work has also suggested the value of using psychological data to estimate similarity. Manning and Kahana (2012) compare latent semantic analysis to a word-association space generated from free association data (Nelson et al., 2004), and note the importance of using multiple measures to explore semantic clustering effects. Given that a participant’s “true” semantic representation is unknown (and will not align perfectly with any model), simulations with multiple measures can provide a sense of what degree of clustering can be expected from this misalignment (e.g., by generating perfectly clustered data under one measure, and calculating semantic clustering scores using another). More broadly, similarity measures derived from behavioral data can sometimes outperform word embeddings. For example, De Deyne et al. (2016) found that similarity ratings were more highly correlated with a measure from a word association space than word embeddings.

Notably, our work does not explore the role of semantic context: while *whale* and *snake* may be similar in the context of a completely random set of nouns, they may be dissimilar in the context of other animals. Prior work has found that context can have large effects on similarity judgments, such as in the case of Tversky (1977), where the presence of irrelevant alternatives led to a choice reversal in a matching task. However, our model may prove a useful tool for exploring the role of context, for example by seeing how a word pair’s similarity changes when other words in the word list are replaced. When similarity is estimated for word pairs that vary in their semantic context across lists, as is the case for our analysis of the PEERS dataset, the results reflect something akin to prototypical similarity. This is very much like small language models, including word2vec, that have been successful in spite of their inability to model contextual shifts in meaning and poor performance with subordinate meanings of polysemous words. More recent language models, including BERT (Devlin et al., 2019) and other embedding models that serve as the basis for large language models, have been very successful because of their ability to account for shifts in meaning in novel contexts. Future work could explore how and whether changes in semantic context affect semantic clustering in free recall.

Our model is not well suited to assess various mechanistic theories of why semantic clustering occurs, in contrast to (for instance) the rehearsal account (Rundus, 1971; Ward & Tan, 2023). Rather, our model is descriptive, based on a purely probabilistic and descriptive account of shifts in lag between study and recall. The model estimates similarity from the likelihood of this shift. Still, aspects of the model may be psychologically meaningful. We chose to model recall lags using a double-exponential distribution largely because prior work has found that the perceived similarity between stimuli typically decreases exponentially with psychological distance (Roads & Love, 2024; Shepard, 1987). The same does not need to be true for recall lag, which is an indirect measure of “distance” – yet it does work, suggesting that recall lag is a reasonable proxy for psychological distance. Other possible distributions (such as a Gaussian) or different parameterizations of the exponential could be explored that are potentially a better approximation of mental similarity. For example, an asymmetric Laplace function might be more suitable for estimating similarity, accounting for the fact that participants tend to recall words in forward rather than backward order (Kahana, 1996).

In contrast to descriptive models, process models of free recall are common, and have been successful in the literature because they account for a wide range of effects in free recall, including semantic clustering. However, these models make additional assumptions about cognitive mechanisms and representation beyond the shape of a similarity gradient. For example, the context-maintenance and retrieval model (CMR; Polyn et al., 2009) incorporates a semantic representation in the form of a similarity matrix to account for semantic clustering. Polyn et al. (2009) use latent semantic analysis to construct this similarity matrix, but our model can also be used to generate such a matrix to be embedded in a larger and more encompassing model of free recall. (In the Electronic Supplementary Material, we provide the word-pair similarities estimated from the PEERS dataset that can be used for this purpose.) It is plausible (though not yet tested) that similarities from our model could provide a better fit to data than those derived from latent semantic analysis or word-association tasks, because in our model they are derived from free recall data itself. Morton and Polyn (2016) provide a framework for making such comparisons across different semantic models. Alternatively, as Morton and Polyn (2016) suggest, one could estimate the optimal (maximum likelihood) similarity matrix directly within CMR, though this is considerably more complicated than our approach. With these points in mind, the current work provides a novel methodology for estimating semantic similarity from free recall data that could be applied to many potential research questions.

## Footnotes

1 These categories were constructed by Hong et al. (2024) by applying a clustering algorithm to a similarity matrix of a larger word pool. The category labels used here (e.g., leather goods) were not used by Hong et al. (2024) but were chosen by us for descriptive purposes.

2 Relatedness was determined through manual inspection by one of the authors, blind to condition. A list of these is available in the Electronic Supplementary Material.

3 In Fig. 6, we combine networks from both word pools (lists one and two), but since there was no overlap between word pools, no edges can be estimated between words from different word pools. Alternatively, these can be viewed as four distinct networks – one for each word pool by list type.

4 Study lag was included as a predictor in case similarity estimates from our model were inflated from appearing in nearby serial positions. In other words, we wanted to ensure that the results were not driven by temporal similarity.

5 The experiment also included a 24th session with novel words, which we did not analyze.
